# Metagenomic insights into effects of thiamine supplementation on ruminal non-methanogen archaea in high-concentrate diets feeding dairy cows

**DOI:** 10.1186/s12917-018-1745-0

**Published:** 2019-01-03

**Authors:** Fuguang Xue, Xuemei Nan, Yunlei Li, Xiaohua Pan, Yuming Guo, Linshu Jiang, Benhai Xiong

**Affiliations:** 1 State Key Laboratory of Animal Nutrition, Institute of Animal Science, Chinese Academy of Agricultural Sciences, Beijing, 100193 China; 2 State Key Laboratory of Animal Nutrition, College of Animal Sciences, China Agricultural University, Beijing, 100193 China; 3 Beijing Key Laboratory for Dairy Cow Nutrition, Beijing University of Agriculture, Beijing, 102206 China

**Keywords:** Dairy cows, High-concentrate diet, Metagenomics, Non-methanogen archaea, Thiamine

## Abstract

**Background:**

Overfeeding of high-concentrate diet (HC) frequently leads to subacute ruminal acidosis (SARA) in modern dairy cows’ production. Thiamine supplementation has been confirmed to attenuate HC induced SARA by increasing ruminal pH and ratio of acetate to propionate, and decreasing rumen lactate, biogenic amines and lipopolysaccharide (LPS). The effects of thiamine supplementation in HC on rumen bacteria and fungi profile had been detected in our previous studies, however, effects of thiamine supplementation in HC on rumen non-methanogen archaea is still unclear. The objective of the present study was therefore to investigate the effects of thiamine supplementation on ruminal archaea, especially non-methanogens in HC induced SARA cows.

**Results:**

HC feeding significantly decreased dry matter intake, milk production, milk fat content, ruminal pH and the concentrations of thiamine and acetate in rumen fluid compared with control diet (CON) (*P* < 0.05), while the concentrations of propionate and ammonia-nitrogen (NH_3_-N) were significantly increased compared with CON (*P* < 0.05). These changes caused by HC were inversed by thiamine supplementation (*P* < 0.05). The taxonomy results showed that ruminal archaea ranged from 0.37 to 0.47% of the whole microbiota. Four characterized phyla, a number of Candidatus archaea and almost 660 species were identified in the present study. In which *Euryarchaeota* occupied the largest proportion of the whole archaea. Furthermore, thiamine supplementation treatment significantly increased the relative abundance of non-methanogens compared with CON and HC treatments. *Thaumarchaeota* was increased in HC compared with CON. Thiamine supplementation significantly increased *Crenarchaeota, Nanoarchaeota* and the *Candidatus* phyla, however decreased *Thaumarchaeota* compared with HC treatment.

**Conclusions:**

HC feeding significantly decreased ruminal pH and increased the content of NH_3_-N which led to N loss and the increase of the relative abundance of *Thaumarchaeota*. Thiamine supplementation increased ruminal pH, improved the activity of ammonia utilizing bacteria, and decreased *Thaumarchaeota* abundance to reduce the ruminal NH_3_ content and finally reduced N loss. Overall, these findings contributed to the understanding of thiamine’s function in dairy cows and provided new strategies to improve dairy cows’ health under high-concentrate feeding regime.

**Electronic supplementary material:**

The online version of this article (10.1186/s12917-018-1745-0) contains supplementary material, which is available to authorized users.

## Background

Overfeeding of high-concentrate diet (HC) frequently leads to subacute ruminal acidosis (SARA) in modern dairy cows’ production. The influences of SARA include the decrease of ruminal pH, and the accumulation of propionate, lactate, biogenic amines and lipopolysaccharide (LPS) [1, 2]. Meanwhile, SARA changes the stability of ruminal microbial ecosystem, which is composed by bacteria, protozoa, anaerobic fungi and archaea [3, 4]. Since SARA leads to considerable damage in dairy cows, it is necessary to investigate strategies to attenuate SARA. Fortunately, our previous studies found that thiamine supplementation could attenuate HC induced SARA through increasing ruminal pH and the abundance of thiamine-synthesis related bacteria including *Bacteroides, Ruminococcus 1*, *Ruminobacter* etc. [5, 6]. However, these findings mainly focused on bacterial communities. The effects of thiamine supplementation on other microbial communities such as archaea are still limited.

Members of the archaea contribute about 0.3 to 3.3% of the whole microorganism in the rumen [7]. Methanogens are the predominant archaeal populations in ruminal anaerobic digestion processes and methane emission reduction has been a worldwide urgent issue that needs to be solved. Previous studies indicated that HC feeding reduced methane emission through decreasing the ruminal pH, restraining the activity of methanogens, increasing ruminal propionate content and reducing the substrate of methane synthesis [8–10]. However, little information is available on non-methanogen archaea. Non-methanogens occupy a large proportion of ruminal archaea and may play important roles in rumen micro-ecosystem. Non-methanogens have been identified in anaerobic digestion processes and gradually drew more attention in recent years. Most non-methanogen archaea in anaerobic digestion were primarily identified as *Crenarchaeota* [11]. While, a number of non-methanogens which participate in the ammonia-oxidizing metabolism, were classified to the phylum *Thaumarchaeota* [12]. Although certain number of non-methanogens have been identified, the response of non-methanogen archaea to HC induced SARA and thiamine supplementation is still unknown.

The 16S rRNA sequencing method was widely used in archaea study [13–15]. However, because of the technical limitations, many non-methanogen archaea could not be identified. Metagenomic sequencing method provides accurate information on microbial communities, and the metagenomics method has been widely applied in detecting the diversity and functions of rumen and gut microbiota, which may allow us better understanding the rumen microbial ecosystem [16, 17]. Therefore, metagenomic sequencing method was chosen in the present study to investigate the effects of thiamine supplementation on ruminal non-methanogen archaea in HC induced SARA dairy cows.

## Results

### Effects of HC and thiamine supplementation on dry matter intake, milk production, milk quality, ruminal pH, ruminal thiamine content and rumen fermentation parameters

Results of HC and thiamine supplementation on dry matter intake (DMI), milk production, milk quality, ruminal pH, ruminal thiamine content and rumen fermentation parameters have been stated in our previous study [18] and these data are shown in Additional file 1. Only milk fat, milk protein and ammonia-N (NH_3_-N) are shown in Table 1. In brief, DMI, milk production, milk fat, ruminal pH, and concentrations of thiamine and acetate were significantly decreased whereas concentrations of propionate and NH_3_-N were significantly increased in HC feeding treatment compared with CONtreatment (*P* < 0.05). However, these changes caused by HC were inversed by thiamine supplementation (*P* < 0.05). Milk protein content was not affected by HC and thiamine supplementation treatments (*P* > 0.05).

**Table 1:** Effects of high-concentrate diet feeding and thiamine supplementation on milk quality and Ammonia-N content

Item	Experimental treatments			SEM	*P*-value
	CON	HC	HCT		
Milk fat (%)	3.850^a^	3.395^b^	3.680^a^	0.126	0.046
Milk protein (%)	3.110	3.050	3.080	0.061	0.106
Ammonia-N(mg/100 mL)	10.495^b^	13.863^a^	11.377^b^	1.711	0.006

*CON* control diet, *HC* high-concentrate diet, *HCT* high-concentrate diet supplemented with 180 mg thiamine/kg DMI, *SEM* standard error of the mean

^a, b^means within a row with different letters differed significantly (*P* < 0.05)

### Identification of archaea communities

The results of metagenomics sequencing have been stated in our previous study [18], which are also provided in Additional file 2. In general, 12 metagenomic libraries were constructed. Based on quality control methods, all pollution data were removed, and approximately 45,000,000 reads per sample were acquired and a minimum of 130,000 contigs per sample were obtained. Taxonomy analysis of total samples was conducted after metagenomics sequencing to investigate the effects of HC and thiamine supplementation treatments on ruminal archaea. The results are shown in Additional file 3. The relative abundance of ruminal archaea ranged from 0.37 to 0.47% of the whole ruminal microbiota in the present study. Almost 660 species of archaea were identified including four characterized phyla and some Candidatus archaea. Among these phyla, *Euryarchaeota,* mostly composed by methanogens, occupied the largest proportion of the whole archaea. Non-methanogens including *Crenarchaeota*, non-methanogenic *Euryarchaeota*, *Nanoarchaeota* and *Thaumarchaeota* accounted for about 20–30% of the whole archaea.

### Effects of HC feeding and thiamine supplementation on the whole archaea

**Fig. 1:**
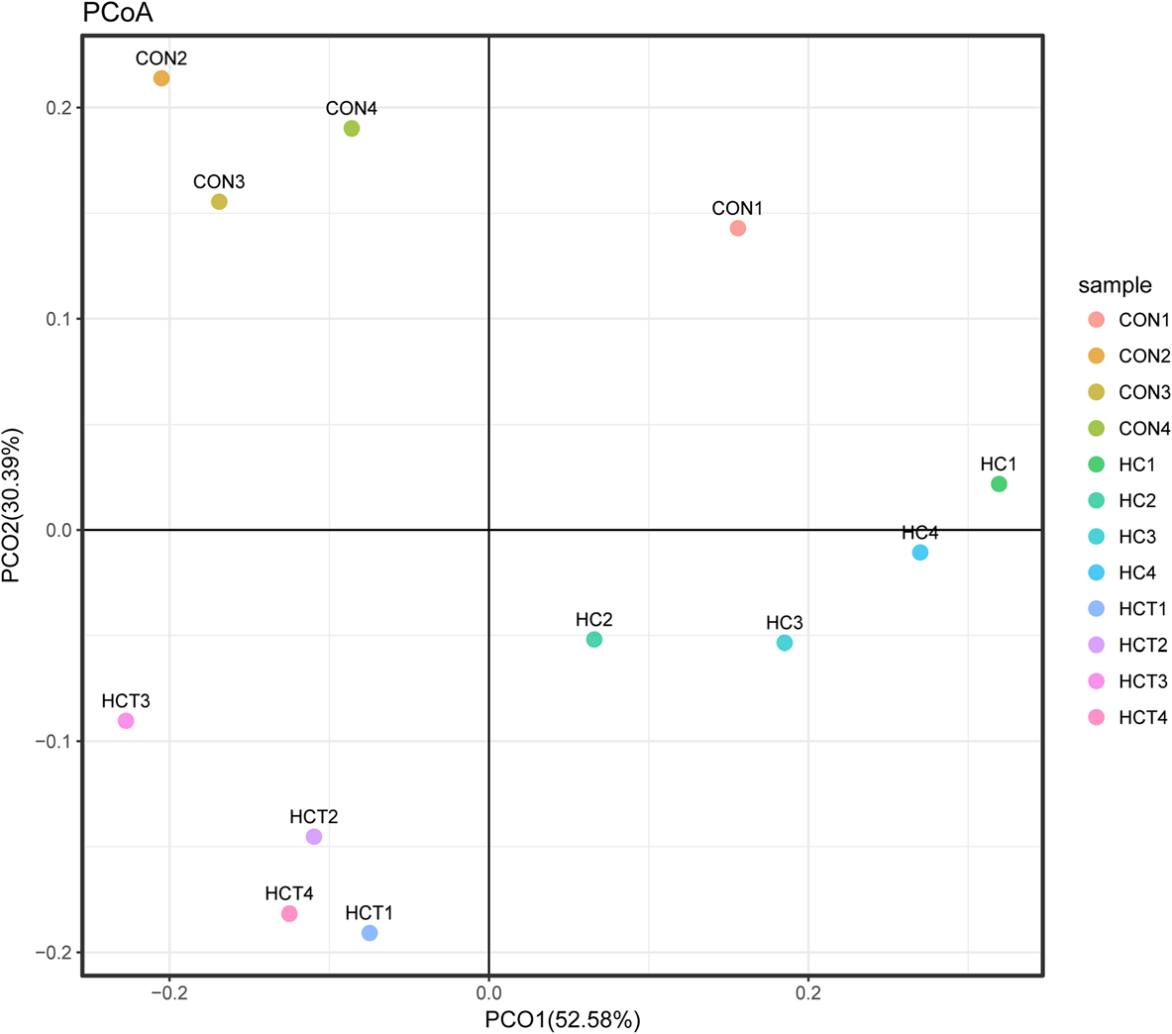
Principal coordinate analysis (PCoA) on community structures of non-methanogens in CON, HC and HCT groups. CON = control diet; HC = high-concentrate diet; HCT = high-concentrate diet supplemented with 180 mg thiamine/kg DMI

Principal coordinates analysis (PCoA) based on unweighted UniFrac distance metrics was conducted to compare non-methanogens profile among the three treatments. As shown in Fig. 1, PCoA axes 1 and 2 accounted for 52.58 and 30.39% of the total variation, respectively. Results indicated that non-methanogens in HC treatment were separated from those in the CON and HCT treatments by PCo1. Non-methanogens in the CON treatment were also separated from those in HCT treatment by PCo2.

Relative abundance of ruminal archaea was then calculated to demonstrate the changes in class or the main species caused by HC and thiamine supplementation treatments. Results are shown in Table 2. HC treatment significantly decreased the total abundance of ruminal archaea compared with CON, while thiamine supplementation increased the total abundance of ruminal archaea, although it was not statistically significant.

**Table 2:** Relative abundances of archaea in rumen fluid from different treatments (%)

Item	Experimental treatments			SEM	*P*-value
	CON	HC	HCT		
*Crenarchaeota*	0.0024^b^	0.0037^b^	0.0217^a^	0.00274	< 0.001
*Euryarchaeota*	0.3849	0.3565	0.3659	0.01353	0.551
*Nanoarchaeota*	0.0002^b^	0.0003^b^	0.0017^a^	0.00023	0.038
*Thaumarchaeota*	0.0035^b^	0.0109^a^	0.061^b^	0.00779	< 0.001
*Candidatus*	0.0036^b^	0.0041^b^	0.0125^a^	0.00108	0.027
Others	0.0023^b^	0.0021^b^	0.0049^a^	0.00045	0.017
total	0.3969	0.3776	0.4677	0.0169	0.063

*CON* control diet, *HC* high-concentrate diet, *HCT* high-concentrate diet supplemented with 180 mg thiamine/kg DMI, *SEM* standard error of the mean

^a, b^means within a row with different letters differed significantly (*P* < 0.05)

### Effects of HC feeding and thiamine supplementation on non-methanogens

As shown in Table 3, total relative abundance of non-methanogens was significantly changed by thiamine supplementation in HC feeding. HC treatment increased the relative abundance of *Thaumarchaeota* and decreased the relative abundance of non-methanogenic *Euryarchaeota* significantly compared with CON. The other phyla were not affected by HC treatment. In contrast, thiamine supplementation significantly increased *Crenarchaeota, Nanoarchaeota* and *Candidatus* phyla, but significantly decreased *Thaumarchaeota* compared with HC treatment. However, non-methanogenic *Euryarchaeota* was not affected by thiamine supplementation.

**Table 3:** Relative abundances of archaea in rumen fluid of different treatments (%)

Item	Experimental treatments			SEM	*P*-value
	CON	HC	HCT		
*Crenarchaeota*	0.0024^b^	0.0037^b^	0.0217^a^	0.00274	< 0.001
Non-methanogenic *Euryarchaeota*	0.0965^a^	0.0782^b^	0.0715^b^	0.006	0.009
*Nanoarchaeota*	0.0002^b^	0.0003^b^	0.0017^a^	0.00023	0.038
*Thaumarchaeota*	0.0035^b^	0.0109^a^	0.061^b^	0.00779	< 0.001
*Candidatus*	0.0036^b^	0.0041^b^	0.0125^a^	0.00108	0.027
Others	0.0023^b^	0.0021^b^	0.0049^a^	0.00045	0.017
total	0.1085^b^	0.0993^b^	0.1733^a^	0.0169	0.023

*CON* control diet, *HC* high-concentrate diet, *HCT* high-concentrate diet supplemented with 180 mg thiamine/kg DMI, *SEM* standard error of the mean

^a, b^means within a row with different letters differed significantly (*P* < 0.05)

**Fig. 2:**
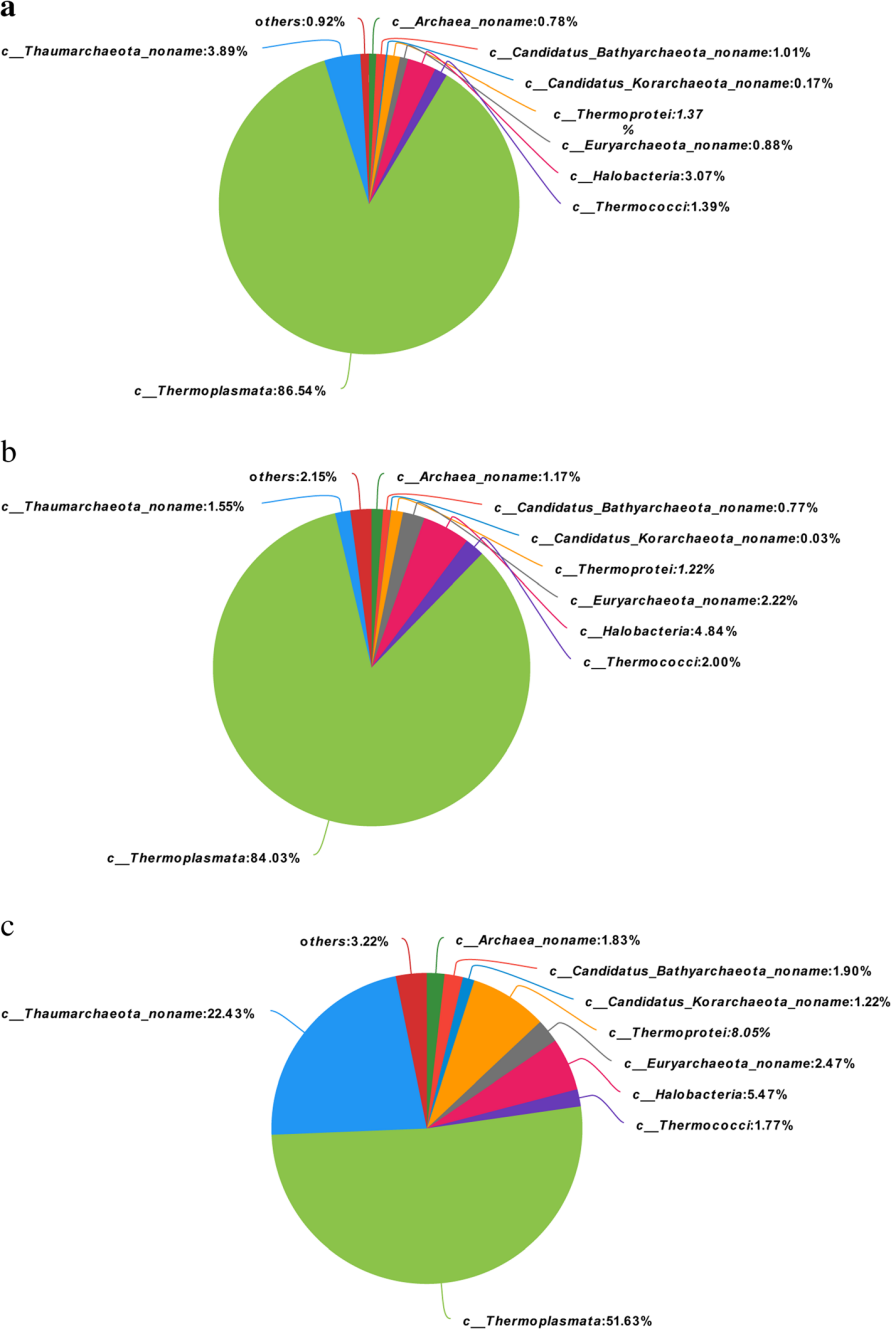
Effects of HC and thiamine supplementation on relative abundances of ruminal non-methanogen archaea on the level of classes. **a** means CON = control diet; **b** means HC = high-concentrate diet; **c** means HCT = high-concentrate diet supplemented with 180 mg thiamine/kg DMI

**Fig. 3:**
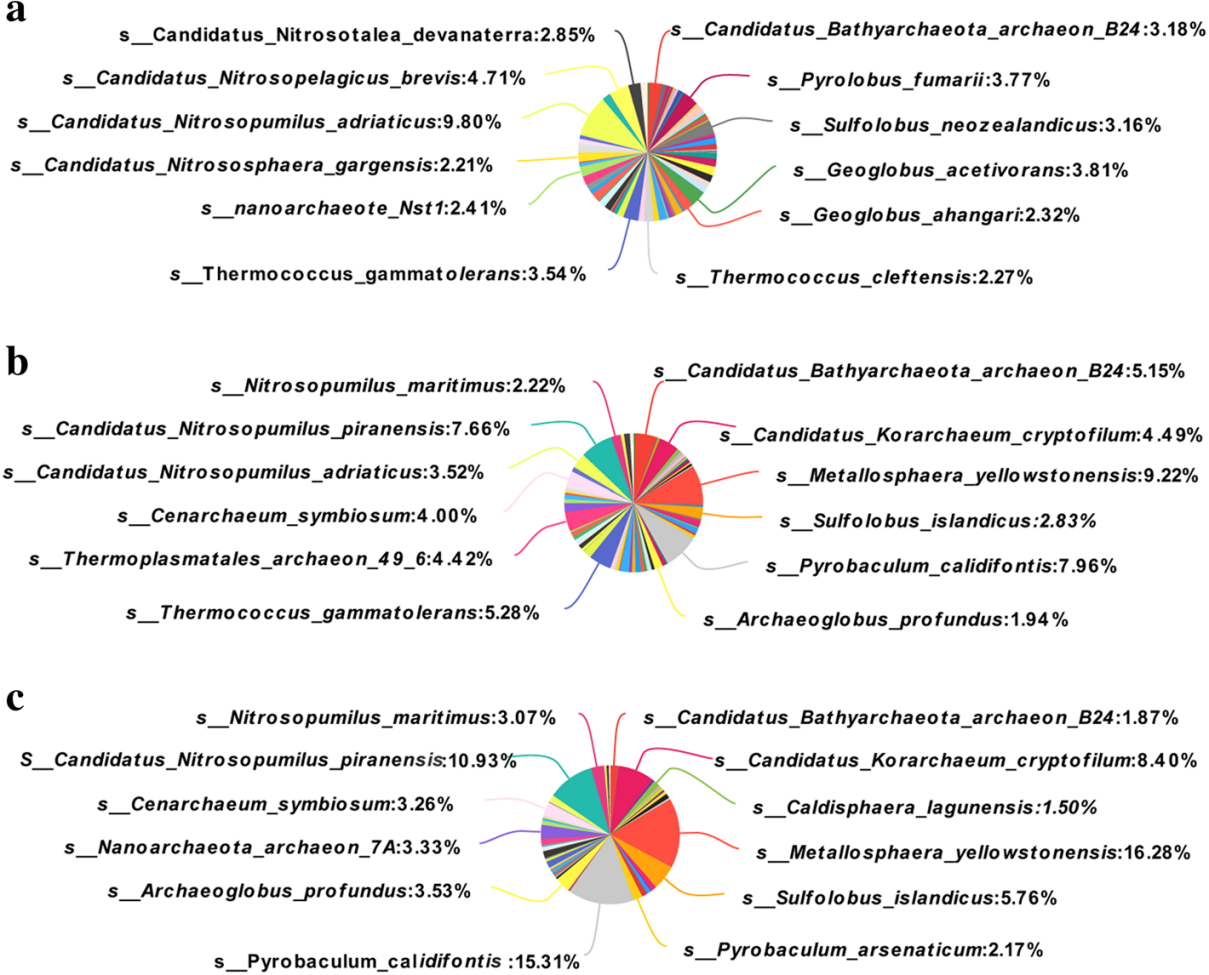
Effects of HC and thiamine supplementation on relative abundances of ruminal non-methanogen archaea on the level of species. **a** means CON = control diet; **b** means HC = high-concentrate diet; **c** means HCT = high-concentrate diet supplemented with 180 mg thiamine/kg DMI

Pieplot analysis was then conducted to investigate the changes of non-methanogen archaea composition. As shown in Fig. 2, at the level of class, *Thermoplasmata* had the highest abundance of non-methanogens in all three treatments. However, the relative abundance of *Thermoplasmata* significantly decreased in thiamine supplementation treatment. While, *Thaumarchaeota_noname* which belongs to the phylum of *Thaumarchaeota* significantly increased in the thiamine supplementation treatment compared with the other two treatments. At the level of species, as shown in Fig. 3, *Nitrosopumilus* was the main species across all three treatments. The relative abundance of species that participated in N metabolism significantly increased in thiamine supplementation treatment compared with HC treatment.

**Fig. 4:**
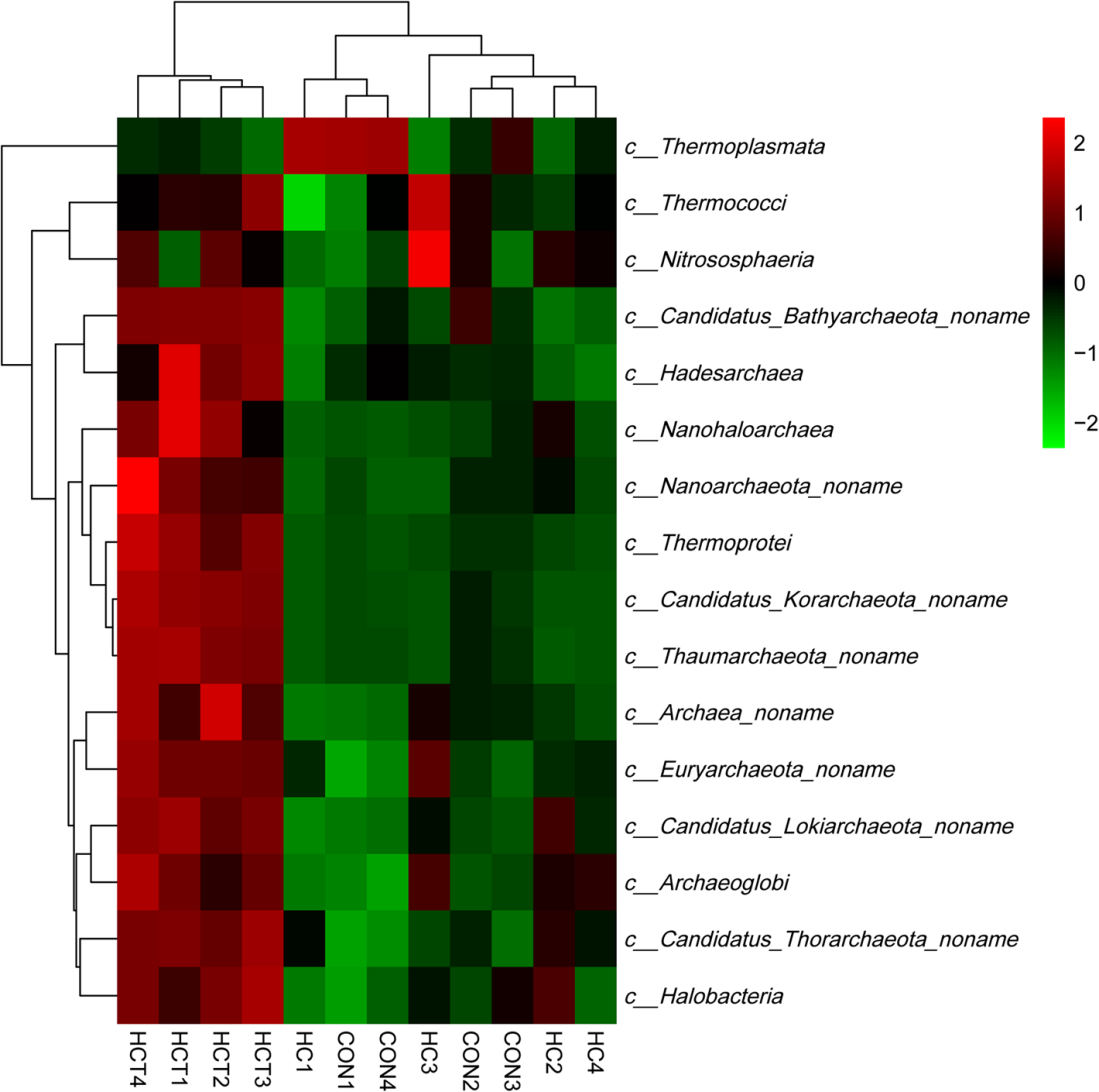
HCA and heat map on relative abundances of non-methanogen class in rumen fluid. CON = control diet; HC = high-concentrate diet; HCT = high-concentrate diet supplemented with 180 mg thiamine/kg DMI

**Fig. 5:**
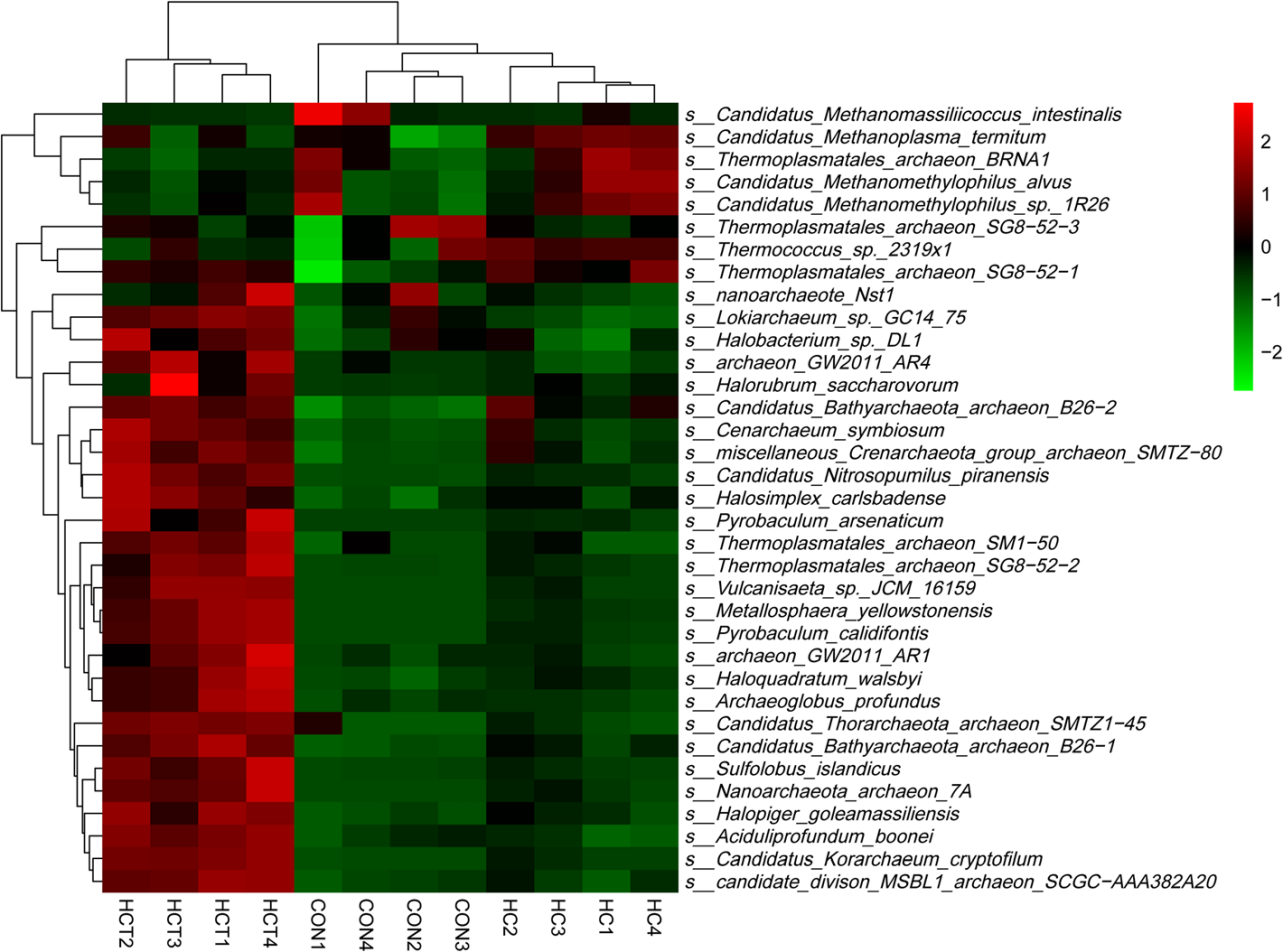
HCA and heat map analysis on the top 35 relative abundances of non-methanogen archaea species in rumen fluid. Rows represent non-methanogens and columns represent samples. Cells were colored based on the relative abundances of non-methanogens in rumen, Red means high abundance; Green means low abundance and Black means the intermediate abundance. CON = control diet; HC = high-concentrate diet; HCT = high-concentrate diet supplemented with 180 mg thiamine/kg DMI

Hierarchical clustering analysis (HCA) and heat map analysis were conducted for further understanding the effects of HC feeding and thiamine supplementation on ruminal non-methanogen archaea profile. Taxonomy level of classes and the top abundance of species were chosen for HCA and heat map analysis. As shown in Figs. 4 and 5, samples of HCT treatment gathered into a cluster which was significantly separated from the other two treatments no matter at the level of class or species. At both level of class and species, *the* abundance of non-methanogens in HCT treatment was significantly higher than that in the other two treatments, except the species in *Thermoplasmata and Thermococci*. Samples of HC treatment were separated from CON treatment at the level of species significantly but not at the level of class. Except *Candidatus_intestinalis*, *Candidatus_termitum*, *Thermoplasmatales_archaeon_BRNA1* and *Candidatus_alvus,* the relative abundances of non-methanogen species were increased in HC compared with CON.

**Fig. 6:**
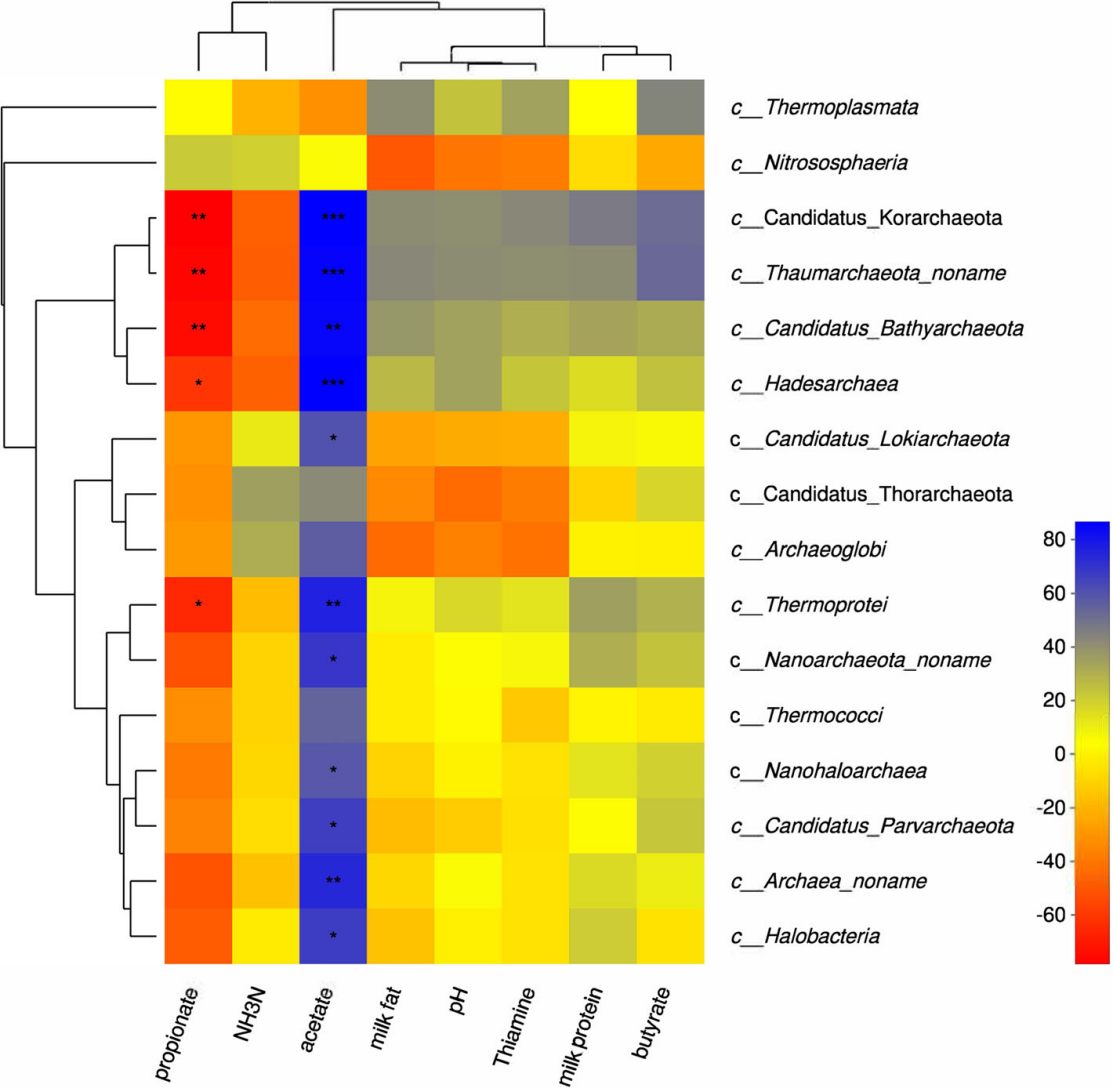
Correlation analyses between relative abundances of non-methanogen archaea and ruminal pH, ruminal VFA, ruminal thiamine, milk fat and milk protein concentrations. Red means a negative correlation; Blue means a positive correlation. “*” means a significant correlation (|r| > 0.55, *P* < 0.05)

In order to demonstrate the correlation between thiamine supplementation and ruminal non-methanogen archaea, Spearman correlation analysis was conducted. As shown in Fig. 6, the relative abundance of all non-methanogen classes (except *Thermoplasmata* and *Nitrososphaeria*) were positively correlated to acetate but negatively correlated to propionate concentration (*P* < 0.05). NH_3_-N content was negatively correlated to *Thaumarchaeota*, *Candidatus Korarchaeota*, *Candidatus Bathyarchaeota* and *Hadesarchaea,* while, milk protein content was positively correlated to these non-methanogens. Furthermore, NH_3_-N content was positively correlated to *Nitrososphaeria*, *Thorarchaeota* and *Archaeoglobi*, Thiamine content had positive relationships with *Thermoplasmata*, *Candidatus Korarchaeota* and *Thaumarchaeota*, however negative relationships with *Candidatus Thorarchaeota* and *Archaeoglobi*.

## Discussion

### Identification of non-methanogen archaea

Non-methanogen archaea, including *Crenarchaeota*, non-methanogenic *Euryarchaeota*, *Nanoarchaeota*, *Thaumarchaeota* and some *Candidatus* phyla were identified in the present study. Many of these non-methanogens have been reported in the microbial communities in marine, hot spring and peatlands [19]. However, it is the first time to be reported in the ruminal micro-ecosystem Methanogens were reported to play important roles in rumen fermentation. Many previous studies have been conducted focusing on the composition of methanogens and the methods of mitigating ruminal methane emission [20–22]. In contrast, non-methanogen archaea were rarely reported. Among the non-methanogens, the discovery of *Thaumarchaeota* in the present study provided new perspectives on the biological diversity and evolution of the archaea in rumen because of its NH_3_-oxidizing function [23]. This could therefore contribute to the understanding of the N metabolism of dairy cattle.

### Effects of HC feeding and thiamine supplementation on the abundance of non-methanogen archaea

The abundances of *Crenarchaeota, Nanoarchaeota* and the whole non-methanogens, were not affected by HC treatment in the present study. This finding was different with those in the studies on ruminal bacteria, fungi and methanogens, which were significantly decreased in HC feeding because of the lowered ruminal pH [6, 10]. The character of extreme environment-tolerance of the non-methanogens could help them defense the decrease of ruminal pH and keep their abundance [24]. Thiamine supplementation increased ruminal pH, improved carbohydrate metabolism and amino acids (AA) metabolism, and consequently provided more available energy [25] which led to the increase of *non-methanogens*.

An interesting finding in the present study was that the relative abundance of *Thaumarchaeota* significantly increased in HC feeding treatment. Its acid-tolerance ability and high N utilization efficiency might be the main reasons for the increasing *Thaumarchaeota* [26]. Characterized as an NH_3_-oxidizing archaea, *Thaumarchaeota* performs the function of the oxidation of NH_3_ to nitrite (NO_2_^−^) in the N cycle [19]. Because of the increased content of crude protein (CP), more N was provided for *Thaumarchaeota* in HC*.* Meanwhile, previous study demonstrated that the optimum pH range for *Thaumarchaeota* was pH < 5.5 [27], and the metabolism activity of *Thaumarchaeota* was promoted by the decreased ruminal pH. Therefore, the relative abundance of *Thaumarchaeota* significantly increased in HC feeding. Because thiamine supplementation increased the relative abundance of ruminal bacteria [6], more N was utilized by the bacteria and less NH_3_-N was released. Thus, the decreased substrates led to the decreased abundance of *Thaumarchaeota*.

### Thiamine, *Thaumarchaeota* and ruminal N metabolism

In the present study, HC feeding significantly increased rumen NH_3_-N content. This was in line with the finding of Mekasha [28] which indicated that HC feeding influenced ruminal N metabolism. In ruminal condition, the N metabolism begins with dietary CP intake, and then CP is hydrolyzed into smaller peptides and AA [29]. The free AA exists short time in ruminal condition because they are utilized by rumen microorganism to synthesize microbial protein. The rest AA are then transferred into urinary N or deaminated to form NH_3_ [30]. The high-concentrate diets, providing more starch and CP, stimulated the proteolytic and amylolytic organisms which usually have cooperation and synergy relationships with each other and consequently resulted in the accumulation of ruminal AA. The decreasing ruminal pH in HC treatment resulted in the decrease of bacterial enzyme activity. Therefore, less AA were used to synthesize into microbial protein and more AA were deaminated into NH_3_ and finally led to the accumulation of NH_3_ [31, 32].

In ruminal and many other conditions, NH_3_ is oxidized by microbiota to form NO_2_^−^, which is the first step in nitrification. This step plays a central role in the global cycling of N. It has been believed for many years that NH_3_ oxidation was conducted only by bacterial species belonging to the classes *Betaproteobacteria* and *Gammaproteobacteria* [33] and assimilated by ruminal bacteria into AA via the reductive amination of glutamate by NAD-linked glutamate dehydrogenase (NAD-GDH) [29, 34, 35]. However, the recent discovery of *Thaumarchaeota* significantly changed the perspective on NH_3_ oxidation [12]. Recent experiments with *Nitrosopumilus maritimus SCM1* indicated that archaea NH_3_ oxidation was dependent on the activity of the NH_3_ monooxygenase enzyme and an unknown enzyme that could convert hydroxylamine (NH_2_OH) to NO_2_^−^ and provide electrons for energy [36]. In ruminal conditions, when rumen pH > 6.0, NH_3_-oxidating bacteria would occupy the predominant status in NH_3_ oxidation process. However, when ruminal pH decreases, especially when pH < 5.5, the NH3-oxidating archaea would play more important roles in NH_3_ oxidation process [27]. In the present study, *Thaumarchaeota* was significantly increased in HC feeding treatment, which confirmed that NH_3_-oxidating archaea played important roles in NH_3_ oxidation process and continually the ruminal N metabolism when pH decreased.

Thiamine supplementation significantly increased the ruminal pH which resulted in the increasing activity of bacterial enzyme to synthesize more microbial protein and reduced the free AA in rumen [25]. It also significantly stimulated the valine, leucine and isoleucine biosynthesis pathways and the protein digestion and absorption pathways, which led to the decrease of NH_3_content in the rumen [25]. On the other hand, the decrease of *Thaumarchaeota* also indicated the decreased NH_3_ content in thiamine supplementation treatment. Thiamine supplementation could therefore decrease the N loss of dairy cattle.

## Conclusion

In summary, HC feeding significantly decreased ruminal pH and increased the content of NH_3_-N which led to the increase of the relative abundance of *Thaumarchaeota* and N loss. Thiamine supplementation in contrast, increased ruminal pH, and improved the activity of NH_3_ utilizing bacteria and *Thaumarchaeota* which could consequently decrease the ruminal NH_3_ content and finally reduce N loss. Overall, these findings contributed to the understanding of thiamine’s function in dairy cows and provided new strategies to improve dairy cows’ health under high-concentrate feeding regime.

## Methods

### Animals, experimental design and dietary treatments

Animals were selected from the dairy cattle farm of the Institute of Animal Science, Chinese Academy of Agricultural Sciences. Animal care and procedures were in accordance with the Chinese guidelines for animal welfare and approved by the Animal Care and Use Committee of the Chinese Academy of Agricultural Sciences. All animals were taken into barrier nursing after the experiment until all animal were recuperated.

Experimental design has been stated in our previous study [18]. In brief, twelve Chinese Holstein dairy cows (627 ± 19.9 kg BW; 180 ± 8 DIM) in second-parity fitted with ruminal cannulas (φ = 10 cm, Bar Diamond, Parma, ID) were randomly assigned into three treatments: a control diet (CON; 20% starch, DM basis), a high-concentrate diet (HC; 33.2% starch, DM basis) and a high-concentrate diet supplemented with 180 mg thiamine/kg DM (HCT, 33.2% starch, DM basis). Thiamine (thiamine hydrochloride, purity ≥99%; Wanrong Science and Technology Development Co., Ltd., Wuhan, China) was added through the rumen cannula. Details of ingredient analysis and chemical compositions of the diets are shown in Additional file 4. Cows were fed twice equally at 06:00 h and 18:00 h each day ad libitum in a single period of 21d. Throughout the experimental period, the cows were housed in individual stalls with free access to fresh water.

### Rumen fluid sampling and parameters measurement

During the experimental period, automatic feeding equipment (Institute of Animal Science Chinese Academy of Agricultural Sciences, Beijing, China, and NanShang Husbandry Science and Technology Ltd. Henan, China) were used to record dry matter intake. Milking facilities of Afimilk (Side-by-Side Parallel Stall Construction, Afimilk, Israel) were applied to record milk production of each cow. Rumen fluid of each cow was sampled at 3 h after the morning feeding on day 21. The rumen fluid was then divided into two potions. One potion was used to analyze the pH value by a portable type pH meter (Testo 205, Testo AG, Lenzkirch, Germany), to detect the thiamine concentration in rumen fluid by high performance liquid chromatography (HPLC, Agilent 1260 Infinity II Prime, Agilent, US) according to Analytical Methods Committee [37] and to measure the rumen volatile fatty acid (VFA) concentrations using a gas chromatograph (GC-2010, Shimadzu, Kyoto, Japan). Concentration of NH_3_-N was determined by indophenol method and the absorbance value was measured through UV-2600 ultraviolet spectrophotometer (Tianmei Ltd., China). The other potion was frozen in the liquid N immediately after adding stabilizer and then stored at − 80 °C for DNA extraction.

### Metagenomic sequencing

Metagenomic sequencing approaches mainly include DNA extraction, library construction, metagenomics sequencing, sequence quality control and genome assembly. The detailed sequencing methods have been stated in our previous study [18]. To state briefly, QIAamp DNA Stool Mini Kit (Qiagen, Hilden, Germany) was used for DNA extraction. TBS-380 and NanoDrop2000 were applied for the measurement of DNA concentration and purity. Paired-end libraries were prepared using TruSeqTM DNA Sample Prep Kit (Illumina, San Diego, CA, USA) and paired-end sequencing was performed on Illumina HiSeq 4000 platform (Illumina Inc., San Diego, CA, USA).

The quality control methods of the reads were stripped using SeqPrep (https://github.com/jstjohn/SeqPrep) [38]. Low-quality reads (length < 50 bp or with a quality value < 20 or having N bases) were removed by Sickle (https://github.com/najoshi/sickle). Reads were aligned to the cow genome by NCBI (https://www.ncbi.nlm.nih.gov) and any hit associated with the reads and their mated reads were removed. The filtered reads were used in assembly by SOAP denovo (http://soap.genomics.org.cn, Version 1.06), which is based on De Brujin graph construction [39]. Scaffolds with a length over 500 bp were retained for statistical tests. The scaffolds were then extracted and broken into contigs without gaps. Contigs were used for further gene prediction and annotation.

### Gene prediction and taxonomy

Open reading frames (ORFs) from each sample were predicted using MetaGene (http://metagene.cb.k.u-tokyo.ac.jp/) [40]. All sequences from gene sets with a 95% sequence identity (90% coverage) were clustered as the non-redundant gene catalog by the CD-HIT (http://www.bioinformatics.org/cd-hit/). Reads after quality control were mapped to the representative genes with 95% identity using SOAP aligner (http://soap.genomics.org.cn/) [16]. DIAMOND method was employed for taxonomic annotations by aligning non-redundant gene catalogs against NCBI NR database with e-value cutoff of 1e^− 5^ and score > 60 [41]. Based on NCBI Microbial Taxonomy Information Database, species annotation information of genes was obtained and relative abundance of species was calculated.

### Statistical analysis

Data were checked for normal distribution using PROC Univariate Normal in SAS 9.2 (SAS Institute Inc., Cary, NC). Differences of ruminal pH, rumen thiamine, rumen VFAs and rumen NH_3_-N concentrations were analyzed using PROC GLM of SAS 9.2. *P*-value < 0.05 was considered as significance and 0.05 ≤ *P* < 0.10 was considered as a tendency. Barplot, pieplot, principal coordinate analysis (PCoA), hierarchical clustering analysis (HCA) and heat map for different rumen non-methanogens were conducted using R package version 3.3.1. Spearman correlation analysis between non-methanogens and ruminal fermentation variables or thiamine content was assessed using the PROC CORR procedure of SAS 9.2. A correlation matrix was created and visualized in a heatmap format using R package version 3.3.1. The abundances of non-methanogens and ruminal variables were considered to be correlated with each other when the absolute values of correlation coefficients (r) were above 0.55 and *P*-values were below 0.05.

### Nucleotide sequence accession number

All the raw sequences were submitted to the NCBI Sequence Read Archive (SRA; http://www.ncbi.nlm.nih.gov/Traces/sra/) under accession number SRP144478.

## Additional files

Additional file 1: Effects of high-concentrate diet feeding and thiamine supplementation on average dry matter intake (DMI), milk production, ruminal pH, ruminal thiamine content and ruminal VFAs content. In this file, the dry matter intake (DMI), milk production, milk fat and protein of each treatment are shown. The ruminal pH, and the ruminal fermentation parameters such as VFAs and Ammonia-N and the thiamine content in each treatment are also shown in this file. (DOCX 19 kb)

Additional file 2: Quantitative information and quality control of sequencing. Metagenomics sequencing information are given in this file. The numbers of total reads we obtained, the reads length, numbers of Contigs, numbers of predicted genes and GC content are shown in this file. (DOCX 18 kb)

Additional file 3: Taxonomy results of ruminal non-methanogen archaea of each samples. Taxonomy results in the level of phylum, class, order, family, genus and species of non-methanogen archaea of each cows are shown in this file. (XLSX 125 kb)

Additional file 4: Ingredient and chemical composition of the experimental diets. In this file, the ingredient and chemical composition of both control diet(CON)and high-concentrate diet(HC) are given. (DOCX 18 kb)

## Abbreviations

AA: Amino acids
BW: Body weight
CON: Control diet
DIM: Day in milk
DMI: Dry matter intake
HC: High-concentrate diet
HCA: Hierarchical clustering analysis
HCT: High-concentrate diet with thiamine supplementation
LPS: Lipopolysaccharide
NAD-GDH: NAD-linked glutamate dehydrogenase
NH_2_OH: Hydroxylamine
ORFs: Open reading frames
PCoA: Principal coordinate analysis
SARA: Subacute ruminal acidosis
SEM: Standard error of the mean
TVFA: Total volatile fatty acid
