# Supplementary material for: Metagenomic insights into effects of thiamine supplementation on ruminal non-methanogen archaea in high-concentrate diets feeding dairy cows
Source: BMC Vet Res. 2019 Jan 3;15:7. doi: 10.1186/s12917-018-1745-0 (PMC6318914; doi:10.1186/s12917-018-1745-0)
Supplement: Supplementary file 1 — Effects of high-concentrate diet feeding and thiamine supplementation on average dry matter intake (DMI), milk production, ruminal pH, ruminal thiamine content and ruminal VFAs content. In this file, the dry matter intake (DMI), milk production, milk fat and protein of each treatment are shown. The ruminal pH, and the ruminal fermentation parameters such as VFAs and Ammonia-N and the thiamine content in each treatment are also shown in this file. (DOCX 19 kb) [file 12917_2018_1745_MOESM1_ESM.docx]

Table S2. Effects of high-concentrate diet feeding and thiamine supplementation on average dry matter intake (DMI), milk production, ruminal pH, ruminal thiamine content and ruminal VFAs content.

| Item | Experimental Treatments | | | SEM | *P*-value |
| --- | --- | --- | --- | --- | --- |
|  | CON | HC | HCT |  |  |
| Average Daily DMI (kg/d) | 21.68^a^ | 19.07^c^ | 20.78^b^ | 0.278 | 0.014 |
| Average Daily Milk production (kg/d) | 27.28^a^ | 22.12^c^ | 23.28^b^ | 1.894 | 0.001 |
| Milk fat (%) | 3.850^a^ | 3.395^b^ | 3.680^a^ | 0.126 | 0.046 |
| Milk protein (%) | 3.110 | 3.050 | 3.080 | 0.061 | 0.106 |
| Ruminal pH | 6.45^a^ | 5.58^c^ | 6.12^b^ | 0.194 | 0.016 |
| Ruminal Thiamine (ug/L) | 16.16^a^ | 9.51^c^ | 13.53^b^ | 1.933 | <0.001 |
| Acetate (mmol/L) | 43.237^a^ | 42.619^b^ | 44.076^a^ | 1.273 | 0.038 |
| Propionate (mmol/L) | 12.899^b^ | 13.849^a^ | 11.846^c^ | 0.632 | 0.027 |
| Butyrate (mmol/L) | 10.770 | 10.354 | 10.819 | 0.137 | 0.356 |
| TVFA (mmol/L) | 70.323 | 70.983 | 70.487 | 1.836 | 0.156 |
| Ammonia-N(mg/100mL) | 10.495^b^ | 13.863^a^ | 11.377^b^ | 1.711 | 0.006 |

a,b,c means within a row with different letters differed significantly (P < 0.05); SEM, standard error of the mean.

CON (control diet); HC (high-concentrate diet); HCT (high-concentrate diet supplemented with thiamine; TVFA, total volatile fatty acid.
