# Supplementary material for: Metagenomic insights into effects of thiamine supplementation on ruminal non-methanogen archaea in high-concentrate diets feeding dairy cows
Source: BMC Vet Res. 2019 Jan 3;15:7. doi: 10.1186/s12917-018-1745-0 (PMC6318914; doi:10.1186/s12917-018-1745-0)
Supplement: Supplementary file 2 — Quantitative information and quality control of sequencing. Metagenomics sequencing information are given in this file. The numbers of total reads we obtained, the reads length, numbers of Contigs, numbers of predicted genes and GC content are shown in this file. (DOCX 18 kb) [file 12917_2018_1745_MOESM2_ESM.docx]

Table S3. Quantitative information and quality control of sequencing

| Sample | | Total reads | Average Read Length(bp) | Contigs | Prediction Genes | N50 | N90 | | GC content |
| --- | --- | --- | --- | --- | --- | --- | --- | --- | --- |
| CON1 | 38117710 | | 144.14 | 136750 | 243399 | 873 | | 402 | 49.73% |
| CON2 | 46684704 | | 142.97 | 150605 | 261343 | 855 | | 393 | 49.02% |
| CON3 | 60005524 | | 144.36 | 205536 | 355099 | 837 | | 387 | 48.87% |
| CON4 | 49418758 | | 144.54 | 185420 | 305814 | 882 | | 426 | 51.60% |
| HC1 | 41149472 | | 142.33 | 130666 | 258388 | 891 | | 369 | 50.30% |
| HC2 | 45783576 | | 144.8 | 164161 | 360929 | 762 | | 297 | 46.59% |
| HC3 | 66780112 | | 144.91 | 266675 | 539128 | 813 | | 327 | 47.17% |
| HC4 | 38925364 | | 144.97 | 140820 | 280129 | 783 | | 321 | 47.10% |
| HCT1 | 51513440 | | 144.22 | 177350 | 270252 | 897 | | 447 | 41.29% |
| HCT2 | 56614214 | | 143.39 | 180487 | 283991 | 879 | | 423 | 43.58% |
| HCT3 | 44973212 | | 144.28 | 161734 | 254637 | 861 | | 402 | 41.59% |
| HCT4 | 38932060 | | 143.7 | 132493 | 180726 | 867 | | 456 | 36.36% |

CON (control diet); HC (high-concentrate diet); HCT (high-concentrate diet supplemented with thiamine)
