# Supplementary material for: Metagenomic insights into effects of thiamine supplementation on ruminal non-methanogen archaea in high-concentrate diets feeding dairy cows
Source: BMC Vet Res. 2019 Jan 3;15:7. doi: 10.1186/s12917-018-1745-0 (PMC6318914; doi:10.1186/s12917-018-1745-0)
Supplement: Supplementary file 4 — Ingredient and chemical composition of the experimental diets. In this file, the ingredient and chemical composition of both control diet(CON)and high-concentrate diet(HC) are given. (DOCX 18 kb) [file 12917_2018_1745_MOESM4_ESM.docx]

Table S1. Ingredient and chemical composition of the experimental diets

| Items | CON | SAID |
| --- | --- | --- |
| *Ingredients( % of DM)* |  |  |
| Chinese wildrye | 11.0 | 5.0 |
| Corn silage | 34.0 | 20.0 |
| Alfalfa hay | 15.0 | 15.0 |
| Ground corn | 10.0 | 30.0 |
| Soybean meal, 43% CP | 14.0 | 14.0 |
| Cottonseed meal | 5.0 | 5.0 |
| Distillers dried grains with solubles | 5.0 | 5.0 |
| Whole cottonseed | 3.0 | 3.0 |
| Limestone meal | 1.0 | 1.0 |
| Calcium hydrogen phosphate | 0.7 | 0.7 |
| Sodium chloride | 0.5 | 0.5 |
| Premix^1^ | 0.8 | 0.8 |
| *Nutrient composition (% of DM)* | | |
| NE_L_^2^, Mcal/kg | 1.58 | 1.68 |
| CP | 18.16 | 18.10 |
| Starch | 19.95 | 30.82 |
| NDF | 36.18 | 27.61 |
| ADF | 23.43 | 17.72 |
| NFC^3^ | 32.67 | 45.74 |
| Ether extract | 4.61 | 4.20 |
| Ash | 5.04 | 4.35 |
| Calcium | 0.88 | 0.84 |
| Phosphorus | 0.55 | 0.55 |

Note: 1 Premix contained (per kg): 2142mg of Cu (as sulfate); 15428 mg of Mn (as sulfate); 15428 mg of Zn (as sulfate); 28 mg of Co (as chloride); 231 mg of I (as iodate); 57mg of Se (as selenite); 2285000IU of vitamin A; 457000 IU of vitamin D;11400 mg of vitamin E.

2 NE_L_ was estimated according to NRC (2001).

3 NFC = NFC = 100 – (% NDF + % CP + % ether extract + % ash) (NRC, 2001)
